# The Role of Behavioral Friction for Patients and Clinicians in a Text-Based Home Blood Pressure Monitoring Program: A Realist Evaluation

**DOI:** 10.21203/rs.3.rs-9569560/v1

**Published:** 2026-06-09

**Authors:** Akhil Mahant, Jennifer Maguire, Mechelle Sanders, Soumya Sridhar, Kevin Fiscella

**Affiliations:** University of Rochester School of Medicine and Dentistry; University of Rochester Medical Center; University of Rochester Medical Center; University of Rochester Medical Center; University of Rochester Medical Center

**Keywords:** Blood Pressure Monitoring, Ambulatory, Behavioral Medicine, Program Evaluation, Health Plan Implementation, Implementation Science, Text Messaging, Hypertension, Health Communication

## Abstract

**Background:**

Home blood pressure monitoring is a proven strategy for improving hypertension control, but implementation challenges remain. Text messaging offers a potentially low-friction, cost-effective alternative for transmitting readings, yet its effectiveness across diverse populations and settings remains underexplored. This study aimed to evaluate the implementation of a text-based home blood pressure monitoring program in a safety-net primary care setting.

**Methods:**

We conducted a realist evaluation of text-based home blood pressure monitoring processes embedded in a type-2 hybrid stepped-wedge, clustered-randomized implementation trial. We developed a program theory informed by behavioral economics and recruited diverse participants using purposive sampling. Patient and clinician participants in the implementation trial were invited for interviews. 52 patients and 51 clinicians were interviewed. We conducted semi-structured interviews in person or by video. Recorded interviews were transcribed and coded to determine barriers and facilitators of the process and identify context-mechanism-outcome configurations to assess the program theory. We also coded relevant meeting notes.

**Results:**

Patients reported an overall positive experience, with facilitators including perceived ease of use, minimal time burden, and practicality. Barriers to patient use, which were minor and rarely mentioned, included technical challenges, limited digital literacy, and lack of clinician response. Clinicians reported mixed experiences. Facilitators included the physical presence of a hypertension coordinator and adequate technological training. Barriers included cumbersome digital tool navigation, lack of integrated reminders, and clinician preference for familiar workflows. Findings aligned with the initial program theory and highlighted the importance of minimizing behavioral friction for both patients and clinicians.

**Conclusions:**

Text-based home blood pressure monitoring is usable for patients, but clinician engagement depends heavily on workflow integration and data accessibility. Weekly multidisciplinary meetings facilitated adaptations that reduced behavioral friction and helped create a usable system for patients, clinicians, and nursing staff. These findings confirm the critical role of behavioral friction in a text-based home blood pressure monitoring program.

## Background

Home blood pressure monitoring (HBPM) represents an evidence-based and cost-effective approach to improve hypertension control.[1, 2] However, scalable strategies are needed for effective implementation.[3] One implementation challenge is the transmission of blood pressure (BP) readings to the clinician, as Medicare reimbursement requires that patients transmit readings 16 days out of the month.[4] Although many different modes of transmission exist, each faces unique obstacles. Telemonitoring using BP monitors equipped with cellular transmitters is expensive. BP monitors with Bluetooth connectivity are less expensive, but connectivity challenges pose a barrier for many older and less technologically proficient patients.[4, 5] Depending on design, digital tools in general can create behavioral friction for patients and clinicians, impeding adoption.[6]

Behavioral friction is a term used in behavioral economics that has been used to refer to mental resistance that hinders individuals from participating in certain actions or behaviors, specifically in the context of take-up of social program benefits.[7] In this study, we similarly use behavioral or psychological friction to refer to cognitive, procedural, or emotional barriers, but in the context of clinician and patient involvement in HBPM and usage of HBPM tools.

Patient texting of BP readings is an alternative, evidence-based, and potentially low friction strategy for transmitting BP readings.[8, 9] A pilot study of Black patients with hypertension insured through Medicaid or Medicare showed higher uptake of and satisfaction with text-based HBPM (TB-HBPM) compared to an online patient portal.[10] However, additional research is needed to evaluate the effectiveness of TB-HBPM across diverse populations and clinical settings, and to validate the promising findings of prior pilot studies.

Outside the United States, realist evaluations have become a widely used approach in evaluating health promotion interventions.[11–13] A realist evaluation addresses the question: What works, for whom, under what circumstances, and why?[14] To do so, it provides a theory-driven approach to examine the influence of contextual factors on causal processes.[14] Realist evaluations are particularly useful when applied to complex healthcare interventions.[11, 15] Realist evaluation posits that a set or series of outcomes results from specific responses (mechanisms) from human and technological actors within a system operating within a particular context or set of conditions.[14] These mechanisms, in turn, are triggered in a given set of organizational or social circumstances (contexts), i.e., material, psychological, organizational, economic, technical, and cultural. An intervention, policy, or program affects the contexts, triggering mechanisms, and, thus, outcomes. Complex interventions can be examined through a combination of context (C), mechanism (M), and outcome (O), referred to as CMO configuration.[14]

In this study, we designed and deployed implementation strategies to promote the effective adoption of HBPM utilizing an automated texting program. We also developed a program theory or logic model outlining connections between mechanisms and processes, allowing us to generate preliminary hypotheses that could be assessed using realist evaluation.

## Methods

### Realist Evaluation

We conducted a realist process evaluation of implementation strategies for a TB-HBPM stepped wedge cluster trial (NCT05488795). We followed the steps outlined in the Realist And Meta-narrative Evidence Syntheses: Evolving Standards (RAMESES) quality and reporting Guidelines based on Pawson and Tilley.[14, 16] In Step 1, we developed a program theory or conceptual model for how the TB-HBPM program was hypothesized to work. Next, we collected data on contexts, mechanisms, and outcomes from codes in patient and clinician interview transcripts and meeting notes. For steps 3 and 4, we used a realist analysis to integrate and synthesize our data.

This sub-study is part of a type-2 hybrid stepped-wedge, clustered-randomized trial designed to implement HBPM in primary care. The details of the research project have been previously described.[17] The core component involves the use of an automated bidirectional texting system (Twistle.com) that allows a patient to securely transmit their home BP readings to a central server. Patients were identified and enrolled in the program by the study’s hypertension coordinator based on prior readings or referrals from their primary care clinician (PCC), typically during their visits after approval from the PCC. Patients without a home BP cuff were provided one at no cost. PCCs, including both physicians and nurse practitioners, received training on BP management. Care teams — including PCCs, medical assistants, registered nurses, and care managers — received team training modeled on TeamSTEPPS (Team Strategies & Tools to Enhance Performance & Patient Safety) an evidence-based framework developed by the Agency for Healthcare Research and Quality to improve team performance in healthcare settings.[18] In this workflow, high BP readings were triaged and managed by care coordinators with referral to the PCC as necessary. PCCs could then view graphs of their patients’ home readings in an online portal known as the digital health tab. PCCs could then adjust medications and refer patients with challenging BP to the clinical pharmacist.

The digital health tab was a portal in EPIC that required a separate login. There was a significant delay in the login. After logging in, the PCP had to navigate several clicks to get to the dashboard where they could view the patients home readings. In room instructions on accessing the digital health tab were provided to sites for clinicians to easily reference during patient visits. There was one standardized set of instructions used throughout the project.

PCCs could also use a customized hypertension template that automatically pulled in office readings and laboratory data. This template was not part of the digital health tab, rather it was found in EPIC and could be pulled into office notes for patients with hypertension. The template also facilitated referrals and treatment intensification.

### Program Theory

Our program theory draws heavily from behavioral economics.[19, 20] We hypothesized that perceptions of patients and clinicians would largely depend on behavioral and/or psychological friction that nudge their behavior.[21] We also expected that clinicians would tailor their approach to individual patient factors as they trained to do.

### Realist Hypotheses

- When patients can enter their BP readings into text message hyperlinks (C), they will perceive the program favorably (O) because of ease of use and time (M).
- When clinicians need to engage in multiple steps to view patients' BP readings (C), they will not routinely view their patients' BP readings (O) because it takes away time from patients during their visits (M).
- When primary care clinicians perceive that a patient with uncontrolled BP can reliably perform tasks required for BP self-measurement and texting (C), they tend to refer patients to the program (O), because they perceive greater benefit to the patient (M).
- When the BP coordinator is physically present (C), clinicians will refer patients to the program (O) because her presence will remind them to refer patients(M).
- When a patient is being seen for hypertension management (C), the clinician is more likely to check the patient’s home BP readings (O) because those data will inform in-the-moment clinical decision-making (M).
- When clinicians view multiple home BP readings above 140/90 mm Hg (C), they are more likely to intensify treatment (O) because they have greater trust in the mean BP than single readings (M).
- When clinicians care for older frail patients (C), they often use a higher BP target (O) because they see less benefit and higher risk to the patient (M).

### Data Sources

Patients who participated in the program were purposively invited for semi-structured interviews to ensure a diverse demographic. The interview guide used was developed specifically for this study, see Supplementary File 1 for the full patient interview guide. Open-ended questions addressed patients' experience with both HPBM and the texting program, including usability, problems, and recommendations for improvement. Questions were focused more on patients’ experiences with the steps in the program, rather than hypertension more broadly. Clinicians participating in the TB-HBPM program were emailed to determine their willingness to participate in a brief interview. We purposively sampled from three groups — physician residents, physicians (MD/DO), and nurse practitioners — aiming for representation across gender and level of training or tenure. Those interested were asked a series of open-ended questions regarding training, patient referrals, use of the BP dashboard, the hypertension template, and BP targets. Questions were structured around the sequential steps of the program and explored perceived barriers, facilitators, and usefulness of each component. Interviews were conducted from April 2024 to July 2024. The interview guide was informed by our overall program theory regarding how the intervention was expected to function, for whom, and under what conditions. The interview guide used was developed specifically for this study, see Supplementary File 2 for the full clinician interview guide. Interviews were recorded and transcribed for later analysis and continued until no new themes emerged. We also coded relevant meeting notes to capture the perspectives of nursing and care managers.

### Analysis

All researchers were trained to code interview transcripts using the qualitative data management system, MAXQDA and in realist evaluation and using Context-Mechanism-Outcomes configuration. We began with high-level codes that referenced key components and steps in the TB-HBPM program — such as clinician team and BP training, patient enrollment processes, clinicians accessing HBPM readings, and BP management decisions. These codes, which included barriers and facilitators within each step, were primarily de novo. Team members discussed these codes at biweekly, mostly remote meetings and then considered contexts or conditions that triggered outcomes within these categories. Codes were then organized as Context-Mechanism-Outcome configurations.

### Participant Recruitment and Consent

Verbal informed consent was obtained from all study participants before data collection and audio recording of the interview. Data was deidentified before analysis. All study participants were offered a $50 honorarium for their time.

### Ethical Review

The research was approved by the University of Rochester Institutional Review Board. We purposively sampled clinicians to ensure diversity by type (family physician vs nurse practitioner), and level of training (resident vs faculty).

## Results

Interviews with 52 patients and 51 clinicians were analyzed using realist methods and organized into context–mechanism–outcome (CMO) configurations corresponding to key steps in the TB-HBPM program. Patient and clinician demographics are displayed in Tables 1 and 2 respectively.

The following findings illustrate how contextual factors and associated mechanisms influenced broader workflow and clinical decision-making processes, patient engagement, clinician referral decisions, and clinician interaction with HBPM data and across the program. Key findings are listed in Table 3.

### Workflow Context and Early Program Adaptations

Early implementation revealed several contextual factors that shaped how the TB-HBPM program functioned in practice. Nursing staff reported that their existing workload made it unfeasible to take on responsibilities related to enrolling patients into the program and training them in HBPM. In this context, staff recommended creating a dedicated hypertension coordinator position. Once implemented, clinicians reported that the coordinator facilitated patient enrollment by reducing the burden on clinic staff and streamlining the referral process.

Another early implementation challenge involved the initial volume of elevated home BP readings submitted through the system. Care managers reported that the number of alerts exceeded their capacity to respond efficiently. In response, the team modified triage thresholds and alert management processes to ensure that notifications reflected clinically actionable readings. These adjustments helped create a workflow that was more manageable for staff while maintaining the clinical usefulness of the program.

The customized hypertension documentation template was rarely used by clinicians. Most clinicians reported continuing to use existing documentation templates that they were already familiar with. In this context, clinicians preferred established workflows (mechanism), resulting in limited adoption of the new template (outcome).

The type of training provided (context) affected clinicians’ perceptions of its clinical relevance (mechanism) and whether they applied it (outcome). Some clinicians reported that training focused on BP management was more valuable than team training because it was directly relevant to their clinical work. Others noted that when training was perceived as less pragmatic or not clearly connected to real clinical workflows, it was more difficult to apply in practice.

These findings provide context for subsequent program components, illustrating how initial implementation tasks and workflow constraints shaped the conditions under which multiple program mechanisms operated.

### Patient Engagement with the Text-Messaging System

Patients generally reported positive experiences using the TB-HBPM system. When patients could measure their BP using an automatic device and quickly transmit readings through text message links (context), they perceived the program as easy and convenient (mechanism), which supported continued participation and positive attitudes toward the program (outcome). Patients frequently mentioned the minimal time required to measure and transmit readings as a key facilitator. The automated nature of the system (context) reduced the effort (mechanism) needed to participate (outcome), and text reminders (context) helped prompt patients (mechanism) to take measurements regularly (outcome). Many patients also appreciated the lifestyle tips and educational messages delivered through the system. Nearly all interviewed patients stated they would recommend the program to family members or friends.

Patient barriers to participation were relatively minor and infrequently mentioned. One patient reported difficulty opening hyperlinks embedded in text messages. Another reported intermittent connectivity issues, or challenges related to limited digital literacy. A few participants described occasional technical problems such as error messages when submitting readings. In some cases, patients reported that a lack of direct clinician feedback (mechanism) after submitting readings (context) reduced their sense that the data were being actively used (outcome). However, these issues rarely led patients to discontinue participation in the program.

These findings align with our hypothesis that minimizing behavioral friction through simple and quick text-based BP submission would promote favorable patient perceptions and sustained engagement.

### Clinical Referral Decisions

Clinicians described exercising clinical judgment when deciding whether to refer patients to the TB-HBPM program. When clinicians perceived that a patient with uncontrolled hypertension was capable of reliably performing home BP measurements and transmitting readings through the texting system (context), they were more likely to refer that patient to the program (outcome) because they believed the patient would benefit from participation (mechanism). In contrast, when faced with patients with competing health or social concerns (context), clinicians judged them as too overwhelmed and unlikely to adhere (mechanism) and were less likely to refer these patients to the program (outcome).

The physical presence of the hypertension coordinator also facilitated referrals. When the coordinator was present in the clinic (context), clinicians reported that her visibility served as a reminder to refer patients (mechanism), which increased enrollment in the program (outcome).

These findings support our hypotheses that both perceived patient capability and environmental cues (such as coordinator presence) influence referral behavior by shaping clinicians’ judgments about patient benefit and prompting referrals within the clinical workflow.

### Clinician Use of Home BP Data

Clinician use of HBPM data was shaped by both the usability of the digital health tab within the electronic health record and the clinical context in which the data were accessed, particularly during hypertension-related patient visits where the information could inform real-time decision-making and counseling. When clinicians could successfully access graphical displays of patients’ home BP readings (context), they reported that the additional information supported clinical decision-making and improved counseling during patient visits (mechanism), increasing their use of these data during encounters (outcome). A patient office visit for hypertension (context) prompted clinicians to review home BP readings through the digital portal to assess BP control, affecting real-time BP management (outcome). Graphical displays within the portal (context) prompted clinicians (mechanism) to visually demonstrate BP trends during patient counseling (outcome).

However, several sources of behavioral friction limited routine use of the portal. Access required a separate login and multiple navigation steps including opening a new encounter (context), which clinicians reported made the system less convenient than existing electronic health record tools (mechanism) deterring regular usage (outcome). Specifically, these additional steps discouraged clinicians from reviewing home readings outside of patient visits (when an encounter was already opened). Time constraints between visits further limited opportunities to access the digital health tab outside of scheduled encounters.

Clinicians also noted that the absence of automated reminders (mechanism) within the electronic health record (context) made it easy to forget which patients were enrolled in the program (outcome). Although in-room instructions and repeated use increased familiarity with the system for some clinicians, dense or complex instructions sometimes discouraged use.

These findings align with our hypotheses that behavioral friction from multiple access steps reduces routine use of HBPM data, while immediate clinical relevance during patient visits increases engagement with the system.

### Clinical Decision-Making and Use of HBPM Data

Clinicians reported that the availability of multiple home BP readings influenced their treatment decisions. When clinicians viewed repeated home BP readings above recommended thresholds (context), they reported greater confidence in intensifying antihypertensive therapy (mechanism), as the average of multiple readings was perceived as more reliable than isolated office measurements (outcome).

Clinicians also described adjusting BP targets based on individual patient characteristics. Clinicians reported taking a more cautious approach when treating older or frail patients (context). In these cases, concerns about medication side effects or treatment-related harms (mechanism) influenced clinicians’ decisions to adopt less aggressive BP targets (outcome). In contrast, for patients with comorbidities such as diabetes or chronic kidney disease (context), clinicians perceived higher cardiovascular risk (mechanism) and were more likely to pursue BP targets below 130/80 mmHg (outcome). In some cases, clinicians reported targeting even lower thresholds.

Few clinicians were aware of the HTN template despite multiple mentions at staff meetings, none reported using it.

## Discussion

We implemented a real-world remote HBPM system using institutional resources and a bidirectional texting system, and evaluated the experiences of participating patients and their primary care clinicians using realist methods guided by the principle of behavioral friction.[17] Our findings largely supported the program theory developed at the outset of the study and illustrate how behavioral friction influenced key stages of the TB-HBPM workflow, including patient engagement, clinician referral decisions, clinician access to home BP data, and treatment decision-making.

Patients reported favorably on the TB-HBPM program, particularly regarding its use of texting. Consistent with our program theory that minimizing behavioral friction would facilitate engagement, patients found the texting system easy to use, required little time to complete tasks, and supported consistent participation. Patients appreciated lifestyle tips delivered via text, reminders to take their BP, and options for adherence reminders, all of which aligned with low behavioral friction. Barriers were less frequent and reflected occasional technical challenges and other isolated issues that, in general, did not deter them from recommending the program. Examples include interruption of cellular service or preferring to be able to enter BP readings into the text message conversation without opening a secure, structured field. These results align with previous reports that document patients finding text messaging to be acceptable, practical, and usable.[22–25]

In contrast to patients who reported consistently favorable experiences, clinicians reported more mixed experiences with several components of the program, particularly those that required interaction with electronic health record tools. As we hypothesized, clinicians reported that the hypertension enroller was an essential facilitator to enrollment. She not only reduced the workload on, but her physical presence served as a prompt to refer patients. As hypothesized, clinicians exercised their judgment in determining which patients were suitable for referrals. Also consistent with the initial program theory, clinicians reported that multiple steps and delays in viewing the patient portal discouraged them from viewing patients’ BP readings, particularly outside a visit. Adoption of the hypertension template was worse than anticipated. However, evidence that adoption of new templates is challenging,[26] with factors related to behavioral friction and expected benefit predominating.[27, 28]

Early in the process, nursing input on the workflow impact led us to recruit and train a hypertension coordinator, whose work proved instrumental to our success. Similarly, care managers' experience with initial alerts triggered by high home BP readings prompted the team to adopt feasible, clinically relevant thresholds for these alerts.

Our findings contributed to institutional changes that improved the clinician's usability of patient dashboards for both individual patients and the clinician’s panel of enrolled patients. The institution contracted with a new vendor that offered a clinician-friendly user interface, integrating it into the EHR for accessing individual patient readings and overall patient panel data. These findings underscore the criticality of closing the loop by minimizing clinician behavioral friction when accessing patient home BP readings.

Our findings are limited by the sampling of primary care patients and clinicians from a single institution, as well as the use of a system from a single vendor. Nonetheless, the use of a realist evaluation confirmed hypotheses based on behavioral friction while allowing us to refine our program theory.

These findings have implications for the design of HBPM systems. In the past decade, systems have emerged for the electronic transmission of blood readings from patients' home BP devices to clinicians' offices. These options include using BP devices with cellular transmitters, using Bluetooth to connect devices to cell phones for transmission, manually entering BP into patient portals, including the use of flow sheets and tracking logs, and using text messages.[29–31] These options differ in terms of ability to prompt patients, costs, and patient usability. Our data are consistent with previous reports indicating that patients perceive texting favorably, particularly for ease of use and additional features such as reminders and the ability to provide health information.[32]

However, BP data alone are not sufficient to close the feedback loop between patients and clinicians. Successful systems require a mechanism for clinicians to review and act upon patients’ data.[1] Our findings largely confirm our hypotheses that behavioral friction is paramount for busy primary care clinicians and registered nurses. Factors such as the need for a separate login, multiple navigation steps, and downloading delays strongly hinder clinician access.[33, 34] Similarly, processes that disrupt clinical workflows or generate excessive non-clinically relevant alerts are not feasible in primary care.

## Conclusions

In conclusion, TB-HBPM is acceptable and usable for patients. However, clinician engagement, including reviewing home readings and adjusting medications, depend heavily on workflow integration and data accessibility. Regular multidisciplinary meetings enable adaptations that reduce behavioral friction and create a usable system for patients, clinicians, and nurses.

## Supplementary Material

Supplementary Files

This is a list of supplementary files associated with this preprint. Click to download.

• SupplementaryFile1PatientInterviewGuidePhase2.docx

• SupplementaryFile2ClinicianKeyInformantSurveyInterviewGuideFINAL.docx

## Figures and Tables

**Table 1:** Patient Demographics

Variables	n = 52
**Age**	59.7 +/−11.6
**Sex n (%)**	
Male	16 (30.77%)
Female	36 (69.23%)
**Race Selections (%)**	
Asian	2 (3.85%)
Black or African American	31 (59.62%)
Other	3 (5.77%)
White	16 (30.77%)

**Table 2:** Clinician Demographics

Variables	n = 51
**Sex n (%)**	
Male	17 (27.87%)
Female	44 (72.13%)
**Clinician Type n (%)**	
Attending Physician	23 (37.7%)
Nurse Practitioner	15 (24.59%)
Resident	23 (37.7%)

**Table 3:** Key Study Findings

Program Theory	Key Findings
Patients can easily text BP readings → positive perceptions	Patients reported ease of use and minimal time burden
Multiple portal steps reduce clinician engagement	Clinicians avoided checking readings outside visits
Clinicians refer patients based on perceived suitability	Clinicians exercised judgment in selecting patients
Coordinator presence increases referrals	Visibility served as reminder to refer
Hypertension visits increase data review	Clinicians checked portal mainly during HTN visits
Multiple readings increase treatment intensification	Clinicians trusted averages more than single readings
Clinicians individualize BP targets	Frail/older patients had less aggressive targets

## Data Availability

The datasets generated and/or analyzed during the current study are available from the corresponding author on reasonable request.

## Abbreviations

HBPM: Home blood pressure monitoring
BP: Blood pressure
TB-HBPM: Text-based home blood pressure monitoring
PCC: Primary care clinician
CMO: Context–mechanism–outcome
